# Deep Learning Technology to Recognize American Sign Language Alphabet

**DOI:** 10.3390/s23187970

**Published:** 2023-09-19

**Authors:** Bader Alsharif, Ali Salem Altaher, Ahmed Altaher, Mohammad Ilyas, Easa Alalwany

**Affiliations:** 1 Department of Electrical Engineering and Computer Science, Florida Atlantic University, 777 Glades Road, Boca Raton, FL 33431, USA; balsharif2020@fau.edu (B.A.); aaltaher2018@fau.edu (A.S.A.);; 2 Department of Computer Science and Engineering, College of Telecommunication and Information, Technical and Vocational Training Corporation (TVTC), Riyadh 11564, Saudi Arabia; 3 Electronic Computer Center, Al-Nahrain University, Jadriya, Baghdad 64074, Iraq; 4 College of Computer Science and Engineering, Taibah University, Yanbu 46421, Saudi Arabia

**Keywords:** image-based, American sign language, deep learning, transfer learning, AlexNet, ConvNeXt, EfficientNet, ResNet-50, VisionTransformer

## Abstract

Historically, individuals with hearing impairments have faced neglect, lacking the necessary tools to facilitate effective communication. However, advancements in modern technology have paved the way for the development of various tools and software aimed at improving the quality of life for hearing-disabled individuals. This research paper presents a comprehensive study employing five distinct deep learning models to recognize hand gestures for the American Sign Language (ASL) alphabet. The primary objective of this study was to leverage contemporary technology to bridge the communication gap between hearing-impaired individuals and individuals with no hearing impairment. The models utilized in this research include AlexNet, ConvNeXt, EfficientNet, ResNet-50, and VisionTransformer were trained and tested using an extensive dataset comprising over 87,000 images of the ASL alphabet hand gestures. Numerous experiments were conducted, involving modifications to the architectural design parameters of the models to obtain maximum recognition accuracy. The experimental results of our study revealed that ResNet-50 achieved an exceptional accuracy rate of 99.98%, the highest among all models. EfficientNet attained an accuracy rate of 99.95%, ConvNeXt achieved 99.51% accuracy, AlexNet attained 99.50% accuracy, while VisionTransformer yielded the lowest accuracy of 88.59%.

## 1. Introduction

Throughout history, humans have employed a variety of communication techniques including gesturing, sounds, drawing, writing, and speaking. However, for people with deafness or hearing impairments, sign language is the primary means of communication and interaction with others, breaking down all barriers of hearing loss condition which severely limits their verbal communication. Because of communication barriers, people with these disabilities have fewer opportunities for development. Sign language is a spontaneous non-verbal language expressed by using manual gestures, facial expressions, and body language to convey messages and meaning. These signs may vary from one country to another, although they have some similarities in using sign language. Unfortunately, there is no universal sign language that can be used for all people with hearing impairments around the world [1]. According to the World Health Organization’s (WHO) most recent research, 5% of the world’s population—432 million adults and 34 million children—have disabling hearing loss, not to mention more than 1 billion people are susceptible to hearing loss due to extended and excessive to loud sounds [2]. In fact, people who lose their hearing sense under any circumstances will lose their ability to speak. The enormous number of deafness and hearing loss conditions has garnered the attention of many researchers and developers in the field of speech recognition and other multidisciplinary fields to conduct their study to assist people with hearing impairments. Their goal is to facilitate the daily life of people with hearing disabilities for communication and social interaction with other individuals. Consequently, with the rapidly growing deaf community, building a sign language recognition system using deep learning technology plays a vital role in interpreting sign language to ordinary individuals and the reverse. This system would ease the process of communication between deaf and normal people. As a result, people with hearing impairments will have the opportunity to become more engaged in society, developing social interaction and relationships [3,4]. Nowadays, the sole means for people with hearing impairments to communicate with other ordinary people is through interpreters. However, it is very costly to hire interpreters who have expertise in interpreting for the deaf, because of the limited number of such interpreters. Nevertheless, there are several obstacles in implementing a sign language recognition system to support the deaf and hearing loss community that should be discussed. Firstly, not all hearing-impaired individuals use sign language as a method of communication, which may give them a sense of isolation and depression. Secondly, there are more than 200 different sign languages and dialects from different countries which may delay the process of implementing a sign language recognition system which would be applicable in various countries [4,5]. Lastly, not everyone is proficient in using today’s modern technology due to a lack of education and development, which can be neglected. Numerous studies and research should be oriented to address and comprehend the obstacles that deaf and hard-of-hearing people encounter, which hinder their societal engagement [6]. The pivotal contribution of our research paper can be illustrated as follows:

- We design a scheme based on deep learning technology to enhance the classification of American Sign Language Alphabet hand gestures.
- We fit five deep learning models to classify and recognize hand gestures with subtle disparities in shape. These models included AlexNet, ConvNeXt, EfficientNet, ResNet50, and VisionTransformer.
- We evaluate the performance of our scheme in terms of accuracy, precision, recall, and F1-score.
- Our scheme outperformed the recent studies utilizing the same dataset.

This research paper is organized as follows: Section 2 provides background information about machine and deep learning techniques. Section 3 highlights relevant research and various methods of deep learning used in sign language recognition systems. Section 4 expounds upon the methodology employed in this study, elucidating the approach and techniques utilized. Section 5 is devoted to the results and discussion of using five different deep learning models. Finally, we present a general conclusion in Section 6.

## 2. Background

The Automated Sign Language Recognition System (SLRS) garnered significant attention from researchers and developers in recent years. This collaborative research area facilitates the construction of sign language recognition systems aimed at supporting individuals with hearing impairments in overcoming communication barriers. In the realm of sign recognition systems, numerous machine and deep learning algorithms have been developed to enable effective communication between individuals with hearing impairments and others. To provide readers with a comprehensive understanding of these algorithms, it is imperative to present a concise introduction to the principles and technologies underlying machine and deep learning. By exploring the foundations of these technologies, readers will gain the necessary background knowledge to comprehend the subsequent discussions on sign language recognition systems.

### 2.1. Machine Learning

**Figure 1:**
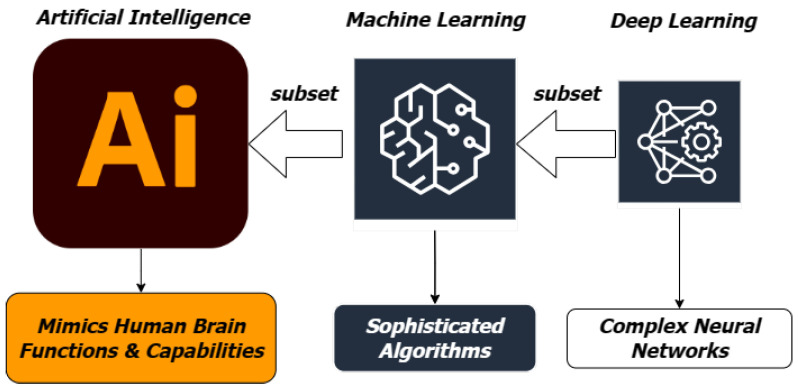
Machine learning is a subset of AI, while deep learning is a subset of ML.

Artificial intelligence (AI) is the process of making machines as intelligent as the human brain to carry out a variety of advanced functions. Machine learning (ML), a subset of AI, focuses on developing computer algorithms capable of learning from data and improving performance through experience. Deep learning, on the other hand, represents a specialized field within ML that utilizes neural networks with multiple layers to extract high-level representations and features. This hierarchical architecture enables models to achieve exceptional performance in various domains. Figure 1 illustrates the relationship between machine learning and deep learning.

The idea of “machine learning” encompasses a variety of stochastic algorithms that can be applied to make intelligent predictions and engage in problem solving based on data. These predictions and the ability to resolve issues automatically improve as the machine learns and generates knowledge from experience. ML is an area in which experts are developing computer algorithms that can access data and learn on their own without being explicit to human intervention [7]. Dealing with massive amounts of data to analyze and extract valuable information requires extraordinary human effort, but with ML, it only takes a few seconds to analyze these data and obtain accurate outcomes [8]. ML algorithms are widely utilized in numerous applications in different sectors including healthcare, transportation, education, energy, agriculture, industry, and many others. Each of these areas has had transformative growth and remarkable development after implementing ML. For instance, in healthcare, ML shows incredible capabilities in predicting diseases based on medical data sources [9,10].

**Figure 2:**
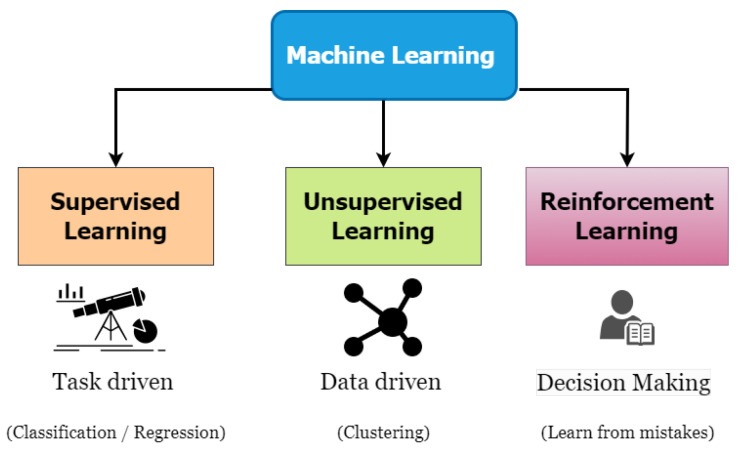
Machine learning classification techniques.

There are three major types of machine learning methods as represented in Figure 2.

#### 2.1.1. Supervised Learning

Supervised learning generates a function that maps a set of input variables x to an output variable y for desired outcomes.

(1) Y=f(X)

These mapping functions can assist in classification and regression. In classification, the model is attempting to predict class labels based on given input data. The input dataset is divided into a training dataset for training the model and a test dataset for testing the model. Regression is related to predicting the numerical value output based on unobservable data. The supervised learning approach is heavily reliant on labeled datasets that can be utilized to train algorithms to accurately classify data or predict outcomes [11,12,13]. The objective of the ML algorithm is to identify patterns and build mathematical models. For instance, by using the supervised learning technique, we may anticipate house prices for the next five years based on already available data, such as x = the size of the house, number of rooms, zip code, age of the house, and current price. y = Predict the price of the house based on the data of x. Many features can be labeled to help algorithms gain knowledge based on previous and present data [14].

#### 2.1.2. Unsupervised Learning

Unsupervised learning is a technique in which users do not have to observe the model. It relies on analyzing and clustering unlabeled data without knowing any information about class labels for input data. Furthermore, it might be challenging to evaluate the outcomes of unsupervised learning techniques. Unsupervised learning algorithms can locate hidden patterns or data groupings, which facilitate the identification of data similarities and differences [11,12]. These abilities to identify hidden and distinct patterns make unsupervised learning an ideal solution for customer segmentation, image recognition, customer persona investigation, anomaly detection, inventory management, and many other applications. Compared to other ML techniques, unsupervised learning can handle more complex tasks.

#### 2.1.3. Reinforcement Learning

Reinforcement learning is a more sophisticated and challenging ML technique. It acquires knowledge via interaction with a set of environments and feedback. It also learns from mistakes [11,12]. RL does not require labeled input/output pairs or examples and sample data due to its dependency on making decisions. Therefore, RL labels should be assigned to each dependent decision [11,12]. RL frequently works with applications requiring fast reactions and adaptation to change under operational conditions. These applications include vehicular traffic management, inventory control, recommender systems, cloud computing, and robotics [15,16,17].

### 2.2. Application and Practices of Machine Learning

**Figure 3:**
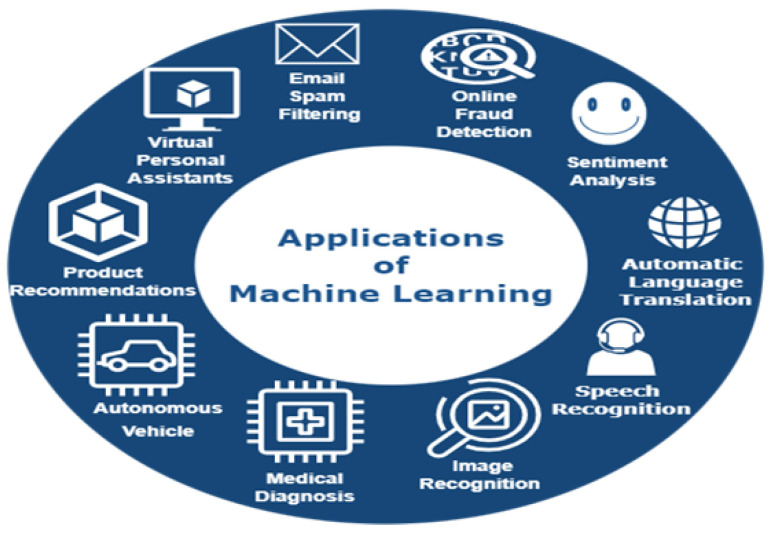
Machine learning applications.

ML is a technique that employs cognitive abilities to allow computers and other technologies to imitate the human brain’s ability to make decisions, solve issues, learn from mistakes, and perform complicated tasks. ML techniques have recently made substantial advancements and offer a promising future in many aspects of our lives. See Figure 3 for a list of existing ML approaches and applications that we use regularly.

### 2.3. Deep Learning

Deep learning is a subset of ML. It is primarily focused on creating large artificial neural network models to process large and complex amounts of data compared with ML techniques [18]. It operates more efficiently with unstructured data. Deep learning can resolve perceptual issues such as problems with images, speech recognition, facial recognition, and handwritten character recognition that computers were formerly unable to handle in the past. These capabilities result from deep learning relying on multiple processing layers for pattern recognition [18,19]. Each layer derives knowledge from the data—the higher the layer level, the more knowledge we can obtain to improve accuracy [8]. The key role of deep learning is to classify, recognize, and describe objects within data. Deep learning automatically extracts features without relying on previous data processing. For the sign language recognition system, deep learning is an excellent method for recognizing and describing hand gestures. Nowadays, deep learning technology is becoming more efficient with real-time applications.

## 3. Related Work

**Figure 4:**
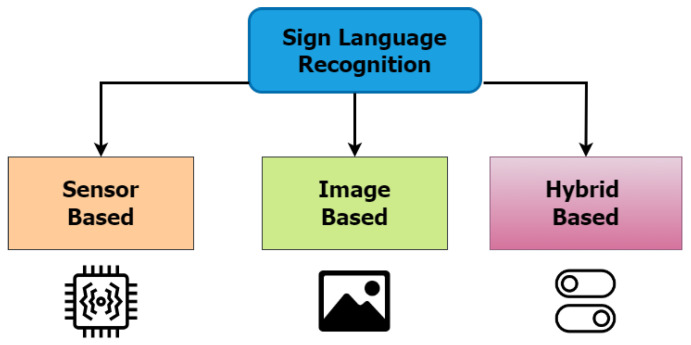
Main approaches for sign language recognition system.

This section explores an influx of related publications on sign language recognition techniques. According to [20,21,22,23], the implementation of a sign language recognition system can be carried out either by using a sensor-based approach, an image-based approach, or both approaches (hybrid), as can be seen in Figure 4.

In the sensor-based system technique, the user wears a specialized glove equipped with multiple sensors and wires. These sensors assist the system in tracking and recording the movements of hands and fingers. The information transmitted to a computer includes data on finger bending, movements, orientation, rotation, and hand position for interpretation. This interaction between the smart glove and computer is a clear example of human–computer interaction. There are two types of sensor-based methods used in sign language acquisition: sensors that can only detect finger bending and sensors that detect hand motion and orientation [23,24]. For more detailed information, we suggest reading the comprehensive survey paper “Systems-based sensory gloves for sign language recognition” [23].

In an image-based approach, there is no need to wear a glove overloaded with wires, sensors, and other materials. The idea behind image-based systems is to use image processing techniques and algorithms to perceive sign gestures [25,26,27]. Image-based sign language recognition systems can be developed using smart devices since most smart devices have high-resolution cameras that allow natural movements and easy availability. Sensor-based systems are accurate and reliable because they simulate hand gestures. Nevertheless, sensor-based techniques have significant drawbacks, such as the user’s heavy glove size making it uncomfortable to wear [20,23,28]. In addition, the glove has several wires connected to a computer, which limits the user’s mobility and its usage of real-time applications [23].

In [29], the authors developed an Arabic sign language (ArSL) recognition system based on a CNN. A CNN is a sort of artificial neural network (ANN) used in deep learning for image processing, recognition, and classification. The system’s implementation recognizes and translates hand gestures into text to bridge the communication gap between deaf and non-deaf people. They used a dataset consisting of 40 Arabic signs, with each sign having 700 different images, which is a principal factor for training systems to have multiple samples per sign. They employed various hand sizes, lighting, skin tones, and backgrounds to increase the system’s dependability. The result showed an accuracy of 97.69% for training data and 99.47% for testing data. The system was successfully implemented in both mobile and desktop applications. In the same context, Ref. [30] introduced an offline ArSL recognition system based on a deep convolutional neural networks model that can automatically recognize letters and numbers from one to ten. They utilized a real dataset composed of 7869 RGB images. The proposed system achieved an accuracy of 90.02% by training 80% of dataset images. The research introduced in [31] aims to translate the hand gestures of two-dimensional images into text using a faster region-based convolutional neural network (R-CNN). Their system mapped the position of the hand gestures and recognized the letters. They used a dataset of more than 15,360 images with divergent backgrounds that were captured using a phone camera. The result shows a recognition rate of 93% for the collected ArSL images dataset. The goal of this proposed study by [32] is to create a system that can translate static sign gestures into words. They utilized a vision-based method to obtain data from a 1080 full-HD web camera of the signer. The camera will capture only the hands to feed into the system. The dataset will be built through continuous capturing. CNN is applied as a recognition method for their system. After training the model and testing it, the system acquired an average of 90.04% accuracy for recognizing the American Sign Language (ASL) alphabet, 93.44% for numbers (from 1 to 10), and 97.52% for static word recognition. In [33], the authors presented a vision-based gesture recognition system that uses complicated backgrounds. They designed a method for adapting to the skin color of different users and lighting conditions. Three types of features were combined: principal component analysis (PCA), linear discriminant analysis (LDA), and support vector machine (SVM) to describe the hand gestures. The dataset used contains 7800 images for the ASL alphabet. The overall accuracy achieved is 94%. The authors in this work [34] utilized a supervised ML technique to recognize hand-gesturing in ArSL using two sensors: Microsoft’s Kinect with a Leap Motion Controller in a real-time manner. The proposed system matched 224 cases of the Arabic alphabet letter signed by four participants, each of whom performed over 56 gestures. The work carried out by [35] presents a visual sign language recognition system that automatically converts solitary Arabic word signs into text. The proposed system has four main stages: hand segmentation, hand tracking, hand feature extraction, and hand classification. The use of the hand segmentation technique is performed to utilize dynamic skin detectors. Then, the segmented skin blobs are used to track and identify the hands. This proposal uses a dataset of 30 isolated words frequently used by hearing-impaired students daily in school. The result shows that the system has a recognition rate of 97%. In [36], the authors created a dataset and a CNN sign language recognition system to interpret the American sign gesture alphabet and translate it to our natural language. Three datasets were used to compare the results and accuracy of each. The first dataset, which belongs to the authors, has 104,000 images for 26 letters of ASL; the second dataset of ASL has 52,000 images; and the third dataset contains 62,400 images. The datasets were split into 70% for the training sets and 15% each for the validation and testing sets. The overall accuracy for all three datasets based on the CNN model is 99% with a slight difference in the decimal values. For another proposed sign language recognition system, ref. [28] trained a CNN deep learning model to recognize 87,000 ASL images and translate them into text. They were able to achieve an accuracy of 78.50%. For another ASL classification task, ref. [37] developed EfficientNet model to recognize ASL alphabet hand gestures. Their dataset size was 5400 images. They achieved an accuracy of 94.30%. In the same context, [38] used the same dataset of 87,000 images for classification. They used (AlexNet and GooLeNet) models, and their overall training results were 99.39% for AlexNet and 95.52% for GoogLeNet. In [39], the authors evaluated ASL alphabet recognition utilizing two different neural network architectures, AlexNet and ResNet-50, using the same dataset that we used. The results showed that AlexNet achieved an accuracy of 94.74%, while ResNet-50 outperformed it significantly with an accuracy of 98.88%.

Table 1 demonstrates list of proposed systems, methods, number of images used in each dataset, and the accuracy rate.

## 4. Methodology

This section explores the five distinct deep learning models for identifying American alphabet gestures. Moreover, it provides comprehensive details on image-based techniques and a brief overview of the dataset employed in ASL recognition systems.

### 4.1. Image-Based Method

Image-based approaches for ASL recognition systems can be categorized into three types: image-based American alphabet signs recognition, American isolated signs recognition, and continuous American signs recognition [28,40].

#### 4.1.1. Image-Based American Alphabet Signs Recognition

**Figure 5:**
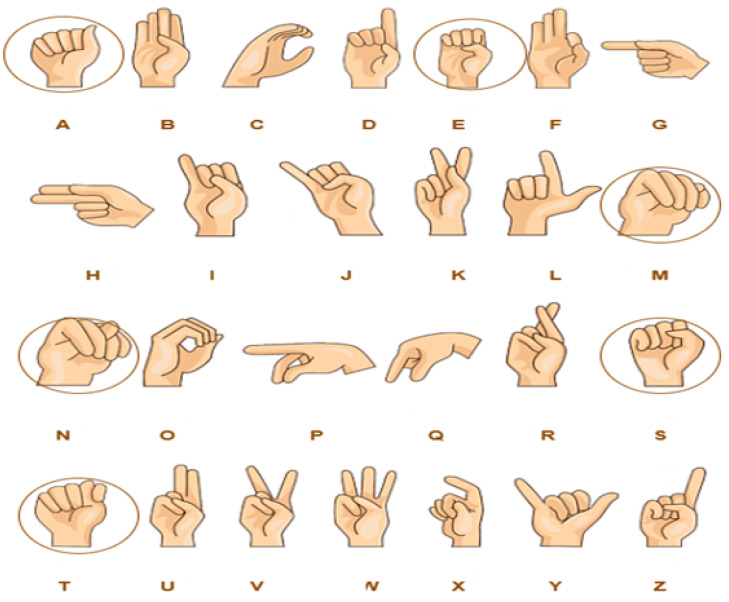
American sign language alphabet and similar alphabet gestures example.

In the alphabet signs recognition system, each letter of the American alphabet is signed separately by the signer using one hand. The deaf community uses alphabet letters to spell people’s names, places, and other words. The semantic meaning of the hand gesture of the alphabet letters comes from its shape, as is the case with the representation of the letters “C”, “D”, “L”, “M”, “N”, “O”, “V”, “W”, and “Z”. In addition, there are some explanations for how other letters are represented that require further investigation. In representing the letters J and Z, the signer must use motion to mimic the shape of each letter. Every image-based recognition system is impacted by visual descriptors, which play a vital role in image processing [41,42]. In fact, there are some similarities between ASL alphabet signs, such as A, E, M, N, and S, which makes it difficult to find a simple deep learning model that can distinguish between hand gestures for classification. The goal of this study was to find a visual descriptor that makes it possible to distinguish between various ASL alphabet gestures. See Figure 5.

#### 4.1.2. Image-Based American Isolated Word Signs Recognition

Isolated word recognition frequently requires a sequence of input images of the entire sign, which is in contrast to alphabet sign recognition. This system works only with letters or words but not complete sentences [28,40]. Image-based isolated words can only handle one word at a time [43].

#### 4.1.3. Image-Based Continuous American Sign Language Recognition

Continuous sign recognition is considerably more complicated compared to the two previous techniques. The primary challenges with this approach are dealing with hand tracking, motion detection, feature extraction, and vocabulary size in a real-time manner [28,40,44]. Many studies have concentrated on developing the most effective features and classification techniques for recognizing and distinguishing between a particular sign from a set of possible ones to accomplish a high accuracy rate.

### 4.2. American Sign Language Dataset

Machine and deep learning, in general, heavily depend on data, as they use algorithms to analyze data and make intelligent predictions. In fact, the availability of sign language databases is limited, which is one of the most significant issues facing sign language recognition and translation systems. Finding a dataset that has manual and non-manual gestures at the same time is challenging [24,40]. Researchers in this field must create a reasonably sized database from scratch to implement and examine their sign language recognition system. Creating a fingerspelling dataset is easy, and it can be performed using non-expert signers to assist in capturing and collecting sign images for the American alphabet with the use of a typical camera. Most letters are depicted in a static posture, and the lexicon is limited to 26 letters. In our proposed system, we only use manual gestures, which represent the American alphabet in sign language. In the finger spelling database, images only display the signer’s hands without any motion; hence, the dataset found in “IEEE Dataport” is suitable for our system. The IEEE Dataport dataset comprises 87,000 images, categorized into 29 distinct classes. Each class encompasses 3000 images, with 26 classes corresponding to the 26 American sign language alphabets, and other classes allocated for space, deletion, and nothing. Dataset images are in RBG format with 200 × 3200 pixels dimensions and different variations [38].

**Figure 6:**
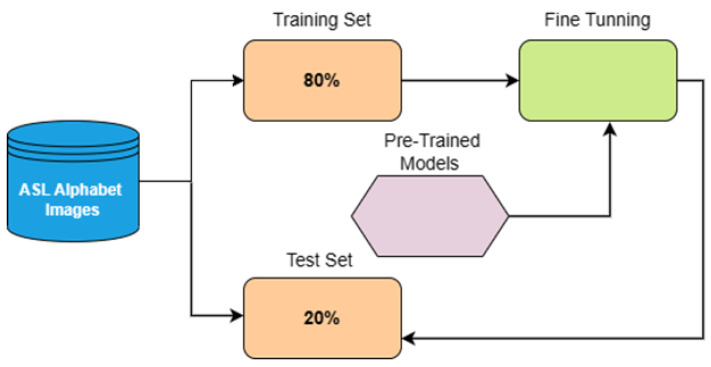
Data pipeline for splitting the ASL Alphabet dataset.

The AlexNet, ConvNeXt, EfficientNet, and ResNet-50 models were trained using 200 × 200 × 3 pixels dimensions, and for VisionTransformer, we resized the dataset to 224 × 224 × 3 pixels. The workflow of splitting the dataset is shown in Figure 6. Then, 80% of the dataset was split into training and validation and split with 5-fold cross-validation approach to train the models. The remaining 20% of the dataset is used for the testing set.

### 4.3. American Alphabet Sign Language Recognition System

The most ordinary form of communication relies on alphabetic expression through speech, writing, or sign language. There is a constant need for a sign language recognition system, as it could reduce the communication gap between those with and without hearing impairments. In this proposed system, we utilized five different deep learning models to produce more effective classification results.

#### 4.3.1. Transfer Learning

In deep learning, the model requires a large amount of data in the training phase to gain more knowledge and skills. However, deep transfer learning is the process of training on a new problem using deep learning models that have already been pre-trained. The core function of transfer learning is to find shared information that can be transferred between various domains. Moreover, it designs suitable algorithms to transfer common knowledge [45]. Transfer learning comprises instance-based transfer, feature-based transfer, and shared parameter-based transfer. See [46] for more detailed information about each approach. In our proposed classification system, we used pre-trained models due to the large amount of data that requires a high amount of computational power for training. Using pre-trained models will accelerate the learning process and save some time [45]. The schematic architecture of a typical sign language recognition system is split into four separate phases:

- Images or video (input data) acquisition;
- Images or video preprocessing;
- Features extraction;
- Classification and recognition of alphabet letters.

**Figure 7:**
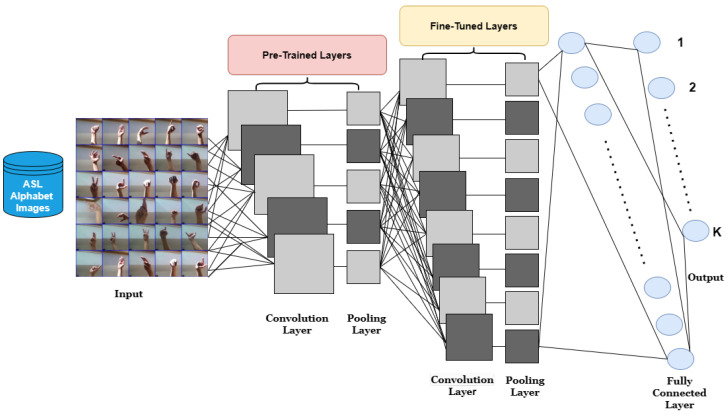
Architecture of transfer learning model.

Using a pre-trained model allows us to exclude some of these required phases. Fine-tuning is applied to the following deep learning models to transfer knowledge to our new tasks. The architecture of the transfer learning model is shown in Figure 7.

A convolutional neural network can be scaled into three key factors when performing image classification:

- The depth indicates the number of layers in the network. Although increasing the depth can help the network learn more intricate features and representations, it can also increase the risk of overfitting and high computational cost.
- The width of a network indicates the number of neurons in each layer. By increasing the width, the representation ability can be improved to recognize fine-grained features.
- The resolution of the input image. By increasing the resolution, the network will be able to capture finer patterns. The drawback of increasing the resolution is that it requires huge memory usage.

#### 4.3.2. AlexNet

**Figure 8:**
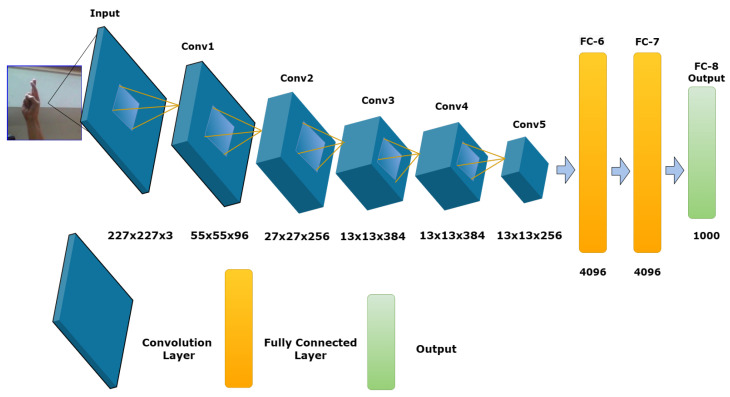
Architecture of the AlexNet model.

The AlexNet model was designed by Alex Krizhevsky, Ilya Sutskever, and Geoffery Hinton. They trained their model on the ImageNet dataset, which contains more than 15 million high-resolution labeled images and 22,000 classes. Their model shows an incredible ability to accurately and efficiently classify more than 1.2 million images. AlexNet uses computing technology called a graphics processing unit to improve image classification performance. The neural network of AlexNet consists of eight layers, including five convolutional layers and three fully connected layers. The number of parameters is 60 million and 650,000 neurons. See Figure 8 for the AlexNet architecture.

**Figure 9:**
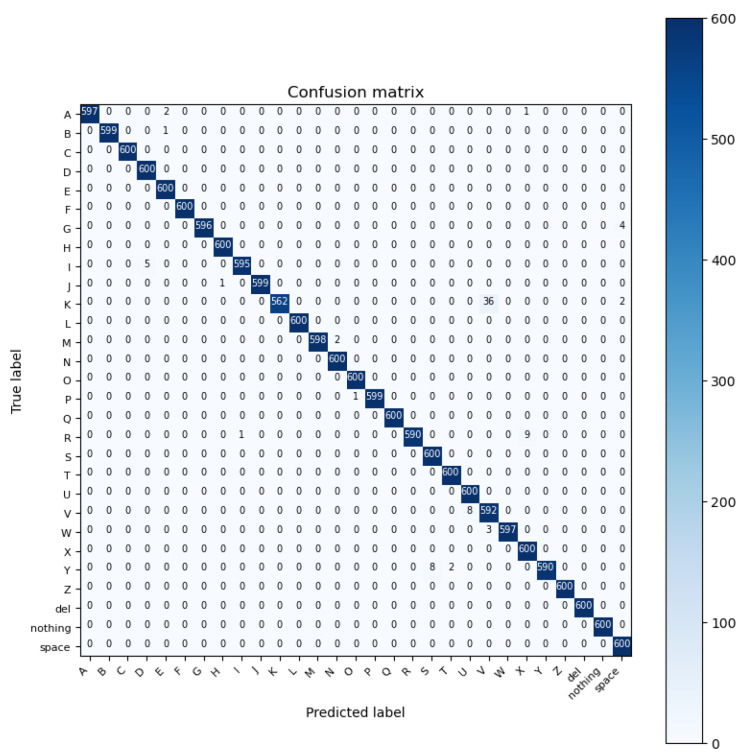
Confusion matrix of the AlexNet model.

Overfitting always happens with larger datasets to avoid or reduce overfitting, and they utilized a developed regularization method known as “dropout” along with rectified linear units (ReLUs), overlapping pooling, and data augmentation [47]. These features of the AlexNet model made it the winner of the 2012 ImageNet Large-Scale Visual Recognition Competition (ILSVRC-2012), an annual image classification competition [48]. Overall, AlexNet played a significant role in advancing the field of deep learning and demonstrated the power of CNNs for image classification tasks [48]. By using the AlexNet deep learning model to recognize ASL alphabet gestures, we were able to train the model and obtain a high accuracy of 99.50% for a dataset of size 200 × 200 × 3. See Figure 9 for the confusion matrix of the result.

#### 4.3.3. Convnext

**Figure 10:**
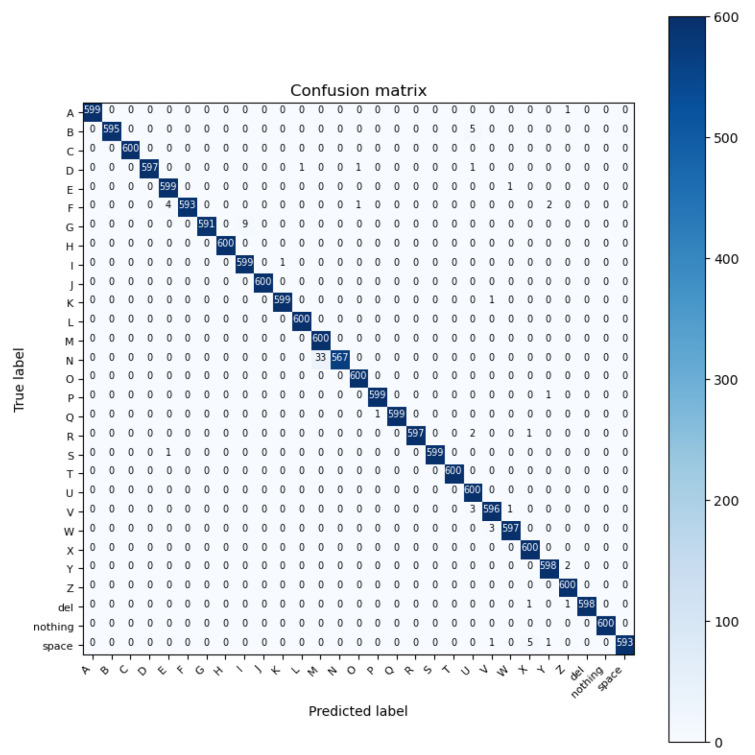
Confusion matrix of the ConvNeXt model.

ConvNeXt is a type of deep neural network architecture created to achieve a state-of-the-art CNN performance on several tasks, including image classification, object detection, and semantic segmentation. ConvNeXt works by processing an input image through a series of convolutional layers and pooling layers, followed by several fully connected layers to classify and recognize, in our case, hand gestures [49]. ConvNeXt helps in recognizing more diverse and complementary features, which leads to better accuracy across a range of image classification tasks. For the ConvNeXt pre-trained model, the accuracy of recognizing the ASL alphabet is 99.51%. The size of the dataset is 200 × 200 × 3 and there is no substantial difference in using AlexNet and ConvNext in terms of accuracy. Figure 10 illustrates the confusion matrix of ConvNeXt.

#### 4.3.4. EfficientNet

**Figure 11:**
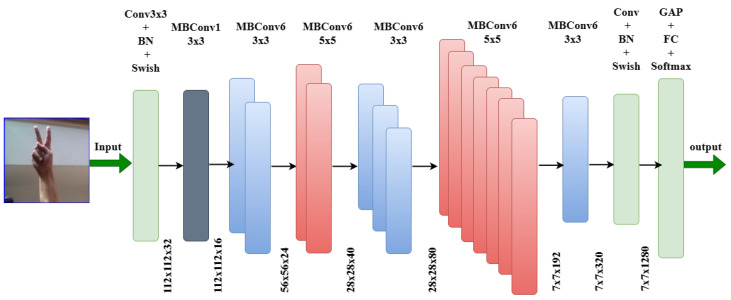
Architecture of the EfficientNet model.

The EfficientNet model is a series of deep neural networks created to use fewer parameters by combining convolutions, bottleneck blocks, depthwise separable, and squeeze-and-excitation modules. EfficientNet uses a set of fixed scaling coefficients to equally scale all dimensions of depth/width/resolution. The rationale behind the compound scaling approach is that larger input images require more layers to expand the network’s receptive area and more channels to identify more fine-grained patterns on the larger image [3]. The models are available in varied sizes and are labeled as EfficieNet-B0, EfficieNet-B1, EfficieNet-B2, etc. The architecture of EfficientNet is shown in Figure 11.

**Figure 12:**
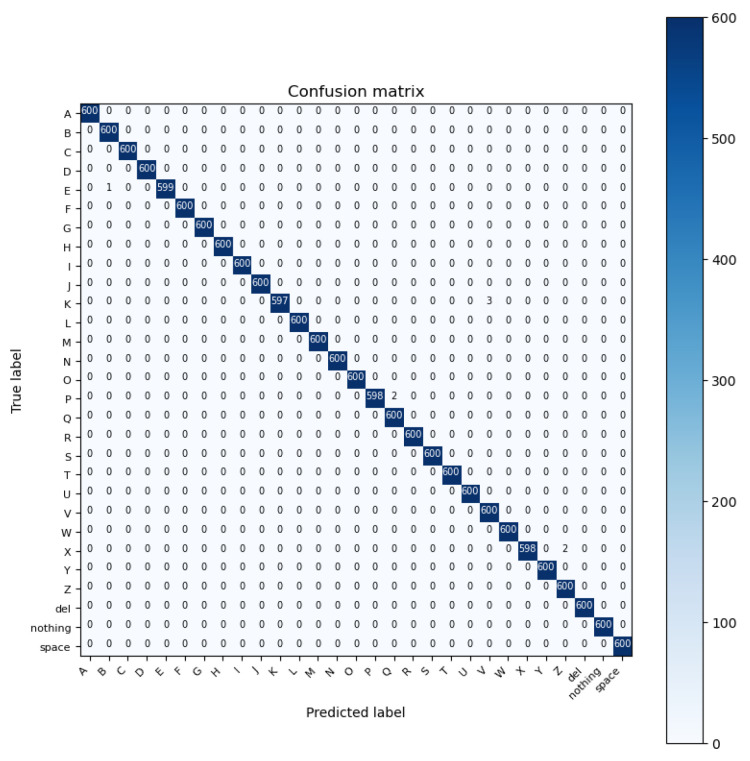
Confusion matrix of EfficientNet model.

Compared with other CNN architecture models, EfficientNet uses less computation time, which leads to less computational cost. An impressive result of 99.95% was obtained using the EfficientNet model to identify hand gestures for the ASL alphabet, and the size of the dataset used is 200 × 200 × 3. Figure 12 illustrates the confusion matrix of the EfficientNet.

#### 4.3.5. Resnet

**Figure 13:**
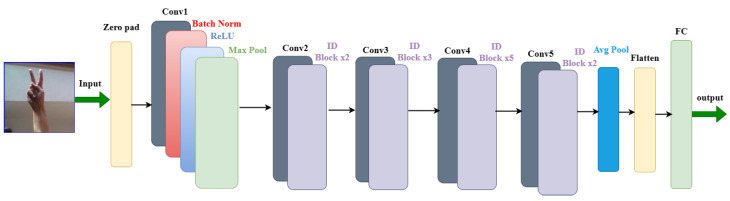
Architecture of ResNet model.

**Figure 14:**
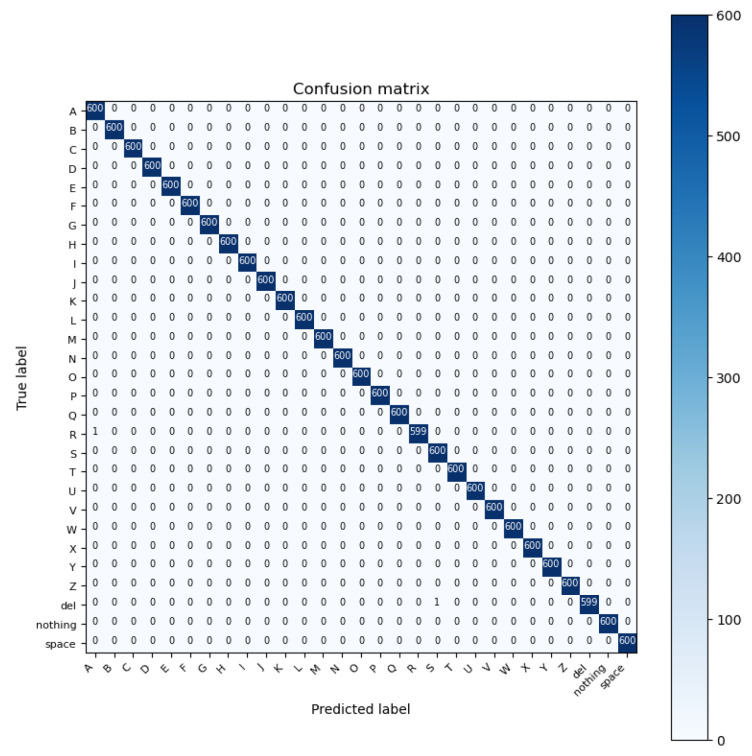
Confusion matrix of ResNet model.

ResNet is a deep learning model which stands for residual network. It is a convolutional neural network (CNN) that was first introduced in the ImageNet Large-Scale Visual Recognition Challenge 2015 (ILSVRC2015) to address the problem of vanishing gradients. ResNet won first place for the LSVRC2015 image classification challenge. The ResNet architecture was designed to support thousands of convolutional layers to avoid vanishing gradients. This is unlike other CNN architectures which are only capable of supporting a few layers, negatively impacting the performance [50]. ResNet’s fundamental concept is to employ “skip connections” to create shortcuts between network layers. These skip connections allow the gradient to flow directly from one layer to another without passing through any non-linear activation functions. ResNet performs with the highest accuracy in recognizing the ASL alphabet 99.98% out of the five deep learning models we examined for image classification tasks. The architecture of the model and the confusion matrix results are shown in Figure 13 and Figure 14, respectively.

#### 4.3.6. VisionTransformer

**Figure 15:**
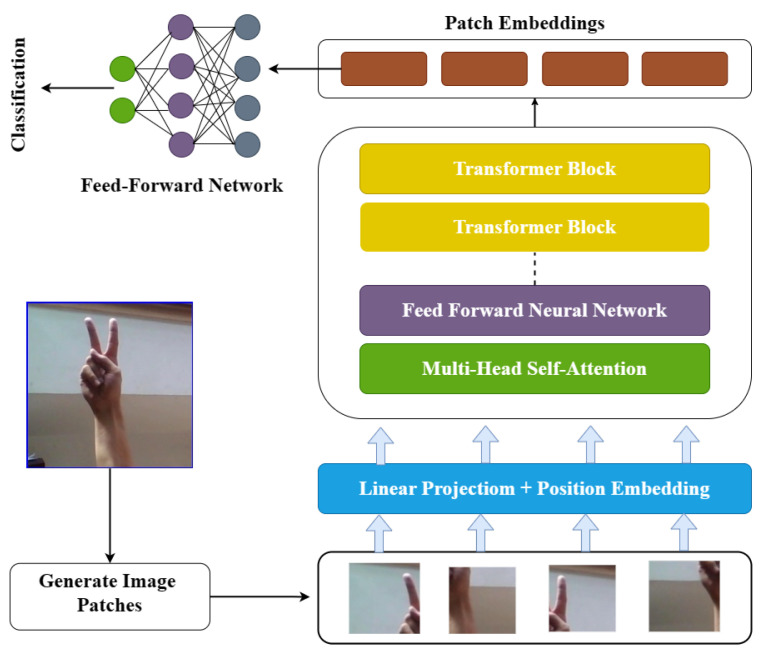
Architecture of VisionTransformer model.

VisionTransformer (ViT) adopts a different strategy from the conventional convolutional neural network (CNN) architecture in image classification. The ViT architecture is composed of two primary components: a patch embedding layer and a Transformer-based encode layer. The patch embedding layer is responsible for converting the input image into a sequence of flattened patches, which are then processed by the encoder. The patches are typically non-overlapping and have fixed sizes of 16 × 16 or 32 × 32 pixels. The encoder is composed of a series of self-attention layers and feed-forward neural networks (FFNs). The self-attention layers allow the model to attend to distinct parts of the input sequence, allowing it to capture long-range dependencies between patches [45]. The FFNs are used to apply non-linear transformations to the output of the self-attention layers. ViT also includes several additional components, such as layer normalization and dropout, to improve the model’s performance and prevent overfitting. ViT is designed to process images in a more flexible and adaptive way than traditional convolutional neural networks by using self-attention [45]. In comparison to CNNs, ViT has a high computational cost, which makes it less useful for some real-time applications see Figure 15 for Vit architecture.

**Figure 16:**
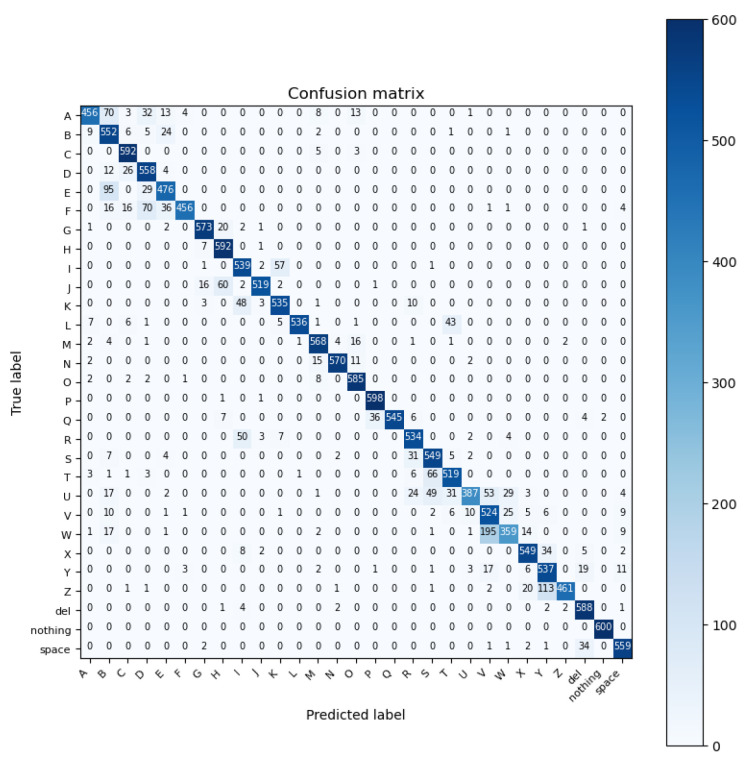
Confusion matrix of VisionTransformer model.

For our task of classifying the ASL alphabet gestures, ViT achieved an accuracy of 88.59%, which is the lowest compared to other deep learning models. This may be related to resizing the dataset to 224 × 224 × 3 to match the model input size requirements. The confusion matrix results are shown in Figure 16.

## 5. Results and Discussion

In the assessment of our scheme, we employed evaluation criteria including accuracy, precision, recall, and F1-score. These metrics are delineated below, based on the factors of true positives (TPs), false positives (FPs), true negatives (TNs), and false negatives (FNs).

(2) $$Accuracy=\frac{TP+TN}{TP+TN+FP+FN}$$

(3) $$Precision=\frac{TP}{TP+FP}$$

(4) $$Recall=\frac{TP}{TP+FN}$$

(5) $$F1=\frac{2*Precision*Recall}{Precision+Recall}=\frac{2*TP}{2*TP+FP+FN}$$

Learning rate is an optimization parameter employed to enhance the performance of a deep learning model. This approach involves adjusting the coefficient, which is responsible for updating the network parameters in response to the error generated during the learning process. In cases where the learning rate is too low, the network parameters are gradually updated, leading to a slower learning process. Conversely, a high learning rate can cause the network to miss the optimal point that minimizes the error. Therefore, optimizing the learning rate is crucial for achieving an optimal performance in the transfer learning models [51,52]. In our image classification task, we employed five different deep learning models that were based on transfer learning and utilized the Adam optimizer. The objective was to train these models to accurately predict American sign language letters and overcome the challenges of distinguishing between similar hand gesture letters. All models were run with the following hyperparameters: a learning rate of 0.001, eight batches, two epoches, Adamax optimizer, and a stochastic gradient descent momentum of 0.9. The five-fold cross-validation was used to measure the performance of the models. Table 2 presents the results obtained from evaluating the performances of the five models. It can be noticed that, in general, ResNet, EfficientNet, AlexNet and Convexnet, respectively, produced the best results in terms of accuracy.

However, the vision transform model exhibited poorer accuracy in comparison to the other models. This can be attributed to the resizing requirement imposed on the input data to conform to the fixed input size of the original model. In order to meet this requirement, the dataset was resized to dimensions of 224 × 3224 × 33. In the resizing process, the potential of losing valuable information exists and that may affect the performance. On the other hand, the other models that attained higher accuracy were trained on the original input size of 200 × 200 × 3.

Applying data augmentation techniques to a vision transformer model, such as random crops, flips, rotations, or adjustments in brightness and contrast, can introduce additional variations to the training data and potentially improve the model’s performance. However, in our specific case, we chose to compare the performance of five models without utilizing data augmentation as a technique across all models. However, to ensure a fair and unbiased comparison between the five models in our study, we refrained from utilizing data augmentation techniques in any of the models. This approach allows us to assess the inherent capabilities and performance differences among the models based on their architectural design and training process without the influence of additional data variations.

Table 3 shows a comparison between the proposed scheme and recent studies that utilized the same dataset [31,37,38,39]. In terms of evaluation metrics for this dataset, our models demonstrate a promising performance. Consequently, the experimental results affirm that the proposed scheme effectively classifies and recognizes hand gestures, even when there are subtle disparities in shape.

Several limitations are encountered when conducting research into deep learning for American sign language (ASL) alphabet classification tasks:

- Dataset availability: The number of datasets containing images for ASL hand gestures is limited due to the need for experts to collect and label data.
- Data diversity and size: Limited availability of diverse and sizable ASL datasets with various backgrounds can hinder the training and generalization of deep learning models, which represented major obstacles when implementing ASL classification task using deep learning models.

## 6. Conclusions

In this paper, we presented a study employing transfer learning techniques utilizing five deep learning models for the effective classification of hand gestures representing the American sign language (ASL) alphabet. The obtained results exhibited remarkable achievements, with the ResNet-50 model outperforming other studies in image classification tasks for ASL recognition systems, achieving an outstanding accuracy rate of 99.988%. Notably, the EfficientNet model demonstrated the second-highest accuracy rate, surpassing 99.95%. Similarly, AlexNet and ConvNext models exhibited commendable accuracy levels of 99.51%. Conversely, the VisionTransformer model exhibited a comparatively lower accuracy rate of 88.59%. This reduced accuracy could potentially be attributed to the preprocessing step involving the resizing of the dataset. In our future work, we plan to convert our trained image classification models for American sign language alphabet gestures into a real-time system and evaluate their performance. We aim to gain insights into their suitability for real-world applications. This will allow us to identify any potential challenges or areas for improvement.

## Figures and Tables

**Table 1:** Comparison of our scheme with recent studies.

Reference	Proposed System	Method	Datasets (Number of Images)	Accuracy
[29]	Smart recognition system for Saudi sign language translate hand gestures into text	CNN	27,301	97.69%
[30]	Recognition System of Arab sign numbers and letters	SVM	7869	90.02%
[31]	Sign language recognition system for Arabic alphabets translate hand gestures into text	ResNet-50	15,360	93.40%
[33]	American sign language recognition system for alphabets	SVM PCA LDA	7800	94%
[36]	American sign language recognition system alphabet signs	CNN	104,000	99.38%
[37]	American sign language recognition system alphabet signs	EfficientNet	87,000	94.30%
[38]	American sign language recognition system alphabet signs	AlexNet GoogLeNet	87,000	99.39% 95.52%
[39]	American sign language alphabet recognition	AlexNet ResNet-50	87,000	94.74% 98.88%
Proposed work	American sign language alphabet recognition	ResNet-50 EfficientNet ConvNeXt AlexNet VisionTransformer	87,000	99.98% 99.95% 99.51% 99.50% 88.59%

**Table 2:** Results of the five models.

Architecture	L.Rate	Optimizer	Accuracy	Precision	Recall	F1
AlexNet	0.001	Adamax	99.50 %	99.51%	99.50%	99.50%
ConvNext	0.001	Adamax	99.51 %	99.51%	99.50%	99.51%
EfficientNet	0.001	Adamax	99.95 %	99.90%	99.94%	99.92%
ResNet	0.001	Adamax	99.98 %	99.95%	99.60%	99.98%
VisionTransformer	0.001	Adamax	88.59 %	89.55%	88.59%	88.54%

**Table 3:** Our proposed method compared to recent studies utilizing the same dataset.

Citation	Year	Models	Accuracy	Precision	Recall	F1
[38]	2019	AlexNet	99.39%	Not reported	Not reported	Not reported
		GoogLeNet	95.52			
[31]	2021	ResNet-18	93.40%	93.30%	94.30%	93.70%
		VGG-16	93.20%	93.60%	93.50%	93.50%
[37]	2022	EfficientNet	94.30%	94.30%	94.46%	94.13%
[39]	2022	AlexNet	93.64%	88.46%	87.88%	87.92%
		ResNet-50	97.41%	94.01%	93.56%	93.88%
Our work	2023	ResNet-50	99.98%	99.95%	99.60%	99.98%
		EfficientNet	99.95%	99.90%	99.94%	99.92%
		AlexNet	99.50%	99.51%	99.50%	99.50%
		ConvNext	99.51%	99.51%	99.50%	99.51%

## Data Availability

Data available upon request.
